# Vaccination with Conserved Regions of Erythrocyte-Binding Antigens Induces Neutralizing Antibodies against Multiple Strains of *Plasmodium falciparum*

**DOI:** 10.1371/journal.pone.0072504

**Published:** 2013-09-10

**Authors:** Julie Healer, Jennifer K. Thompson, David T. Riglar, Danny W. Wilson, Yu-H.C. Chiu, Kazutoyo Miura, Lin Chen, Anthony N. Hodder, Carole A. Long, Diana S. Hansen, Jake Baum, Alan F. Cowman

**Affiliations:** 1 Walter & Eliza Hall Institute for Medical Research, Melbourne, Australia; 2 Laboratory of Malaria and Vector Research, National Institute of Allergy and Infectious Disease, National Institutes of Health, Rockville, Maryland, United States of America; 3 Department of Medical Biology, University of Melbourne, Melbourne, Australia; University of Copenhagen and Rigshospitalet, Copenhagen, Denmark

## Abstract

**Background:**

A highly effective vaccine against *Plasmodium falciparum* malaria should induce potent, strain transcending immunity that broadly protects against the diverse population of parasites circulating globally. We aimed to identify vaccine candidates that fulfill the criteria.

**Methods:**

We have measured growth inhibitory activity of antibodies raised to a range of antigens to identify those that can efficiently block merozoite invasion for geographically diverse strains of *P. falciparum*.

**Results:**

This has shown that the conserved Region III-V, of the *P. falciparum* erythrocyte-binding antigen (EBA)-175 was able to induce antibodies that potently inhibit merozoite invasion across diverse parasite strains, including those reliant on invasion pathways independent of EBA-175 function. Additionally, the conserved RIII-V domain of EBA-140 also induced antibodies with strong *in vitro* parasite growth inhibitory activity.

**Conclusion:**

We identify an alternative, highly conserved region (RIV-V) of EBA-175, present in all EBA proteins, that is the target of potent, strain transcending neutralizing antibodies, that represents a strong candidate for development as a component in a malaria vaccine.

## Introduction

The millennium development goals, set 13 years ago by the United Nations, set a target to halt and begin to reverse the spread of malaria, tuberculosis and HIV by 2015. Coordinated global efforts to roll out insecticide-treated bed nets and improve combination drug therapies have gained significant ground with malaria control measures leading to a reduced global burden of disease and mortality figures have dropped substantially in many areas over the past decade [1]. However, reaching the goal of malaria elimination, and in the longer term its eradication, will require a highly efficacious vaccine.

Immunity that targets the circulating asexual stages of *Plasmodium falciparum,* the causative agent of the most virulent form of human malaria, is the basis of protection from malaria in endemic settings [2]–[4], indicating that a vaccine targeting this stage of the parasite life cycle could provide immunity in susceptible individuals. One favored strategy towards such a vaccine would be to induce immunity that targets the specific process of erythrocyte entry by the blood stage merozoite [5] thus preventing the cycles of intracellular parasite growth and multiplication. The targets of naturally acquired immunity that might facilitate such a strategy, however, are poorly understood.

The process of invasion is mediated by parasite adhesins secreted from the apical region of the infectious merozoite, which bind to receptors on the erythrocyte surface, initiating entry. *Plasmodium* spp. have evolved 2 major super-families of these adhesins, the Erythrocyte-Binding Ligands (EBLs) and the Reticulocyte Binding Ligands (RBLs), that in *P. falciparum* confer the ability to use alternate erythrocyte receptors [6]–[8]. These allow the parasite to access a greater range of host erythrocytes, as well as the ability to circumvent potentially damaging host immune responses (reviewed in [9]). Indeed, in endemic regions, antibodies against EBL and RBL proteins are commonly found in malaria-exposed individuals and have been associated with parasite growth inhibition and protection from clinical malaria [10]–[12] strongly supporting their development towards a vaccine. Whilst individually dispensable for parasite growth *in vitro*, it is clear that parasites require a minimal repertoire of EBL/RBL function to successfully invade [13].

The first identified member of the EBL family, and the best characterized of this group of parasite invasion ligands, is the Erythrocyte Binding Antigen 175 (EBA-175), which binds to sialic acid residues on Glycophorin A, the most abundant protein on the erythrocyte surface [14], [15]. Expressed in the late schizont, EBA-175 is a type-1 transmembrane protein that is housed in the micronemes of the apical complex in merozoites [16], [17]. Around the time of merozoite egress, the micronemes discharge their contents at the apical tip whereupon EBA-175 becomes embedded in the parasite plasma membrane, its extracellular domains exposed to the bloodstream. This exposure occurs “just in time” for binding to erythrocyte receptors following initial contact and reorientation of the merozoite that precedes active invasion. However, it is not known whether EBA-175 exists on the merozoite surface as a single entity, or as part of a larger complex of parasite proteins that interact with the erythrocyte surface. This might involve coordination with other EBL proteins, such as EBA-140 (also known as BAEBL), which binds Glycophorin C, [18]–[20] and facilitates an additional erythrocyte invasion pathway.

The protein structure of EBA-175, and that of all members of the EBL family, is based on the canonical structure defined for the Duffy-Binding Ligand of *P. vivax* [21]. This comprises six extracellular regions (I-VI) followed by a transmembrane domain and a cytoplasmic tail. Common to all EBL proteins are the cysteine-rich domains, regions II and VI. In the *P. falciparum* EBA proteins (EBA-175, −140, and −181), RII is comprised of a tandem Duffy Binding Like (DBL) domain`, with the repeats termed F1 and F2 respectively. Regions III-V (RIII-V) of EBA-175 specifically is dimorphic, with all parasite strains encoding either a C or F allelic haplotypè, based on the original identification from Camp and FCR3 strains [22]. No functional role has yet been identified for this region.

Since all EBL proteins bind erythrocytes through the DBL containing domain [23], [24], vaccine development has focused on RII. In EBA-175 RII has been shown to bind to Glycophorin A both as a single DBL domain [25] and when both DBL domains are present, with the latter shown to interact with sialic acid on Glycophorin A via a molecular handshake [26]. Antibodies against this domain block receptor binding and, to an extent, inhibit invasion *in vitro* [27], [28]. However, RII is also highly polymorphic, showing strong evidence for being under immune selection to maintain genetic diversity in parasite populations [29]. From this perspective, it can therefore be argued that RII may not be an optimal choice for vaccine development, because of the inherent allele-specific nature of a vaccine-induced immune response to this domain.

Among the RBL superfamily in *P. falciparum,* PfRh2a/b and PfRh5 each show promise as potential blood stage vaccine candidates [13], [30]–[33]. PfRh2a/b is a tandem duplicated gene encoding two large proteins that are identical through almost 90% of their sequence, but diverge in the C-terminus [34]. Each binds to an unidentified receptor on erythrocytes through a region in the protein N-terminus [35]. Like EBA-175, there is evidence that Rh2b plays an important role in invasion in some parasite strains [36] and that it is a target of protective immunity to malaria [37]. PfRh5 is unique among *P. falciparum* EBL and RBL superfamily members in that it does not have a trans-membrane domain and is refractory to genetic deletion in every parasite strain tested [30], [31].

Using knowledge of the alternative invasion pathways used by independent *P. falciparum* strains as a first step in identifying candidates for further vaccine development, we expressed eight blood stage antigens from proteins with known roles in erythrocyte invasion, raised antibodies against these antigens, and tested the ability of these antibodies to induce growth-inhibitory responses. We identify EBA-175 RIII-V, as the best performing antigen in this study. As a conserved antigen that induces potent cross strain neutralizing antibodies we propose further development of this domain as a component in a future malaria vaccine.

## Materials and Methods

### Ethics Statement

The culture of *P. falciparum* parasites using donated blood and serum from the Australian Red Cross Society has been approved by The Walter and Eliza Hall Institute Human Ethics Committee (HEC 86/17).

### Antibody generation

Purified EBA175 R2 antigen was kindly provided by Science Applications International Corporation (SAIC) and the PfRh2 N-terminal fragment (15.1) expression construct has been published previously [35]. Sequences encoding the other antigens (Table S1) were codon-optimized for expression in *E. coli* and synthesized by Genscript. 3D7 EBA175 RIII-V, W2mef EBA175 RIII-V and Rh2a/b C terminal Ag were cloned into the pET-45b (+) vector (Novagen) using *Bam* HI and *Xho* I restriction sites to produce recombinant proteins with an N-terminal hexa-His tag. EBA175 RIV-V and 3D7 EBA175 F2-RV were cloned into the pET303/CT-His vector (Invitrogen) to produce recombinant proteins with a C-terminal hexa-His tag. Each plasmid was transformed into BL21 *E. coli* and His-tagged recombinant proteins were purified from soluble lysate by affinity purification on Ni- Sepharose 6 Fast Flow resin (GE Healthcare). Both 3D7 and W2mef EBA175 III-V proteins were further purified by FPLC. Rabbits were immunized with 225 μg and mice with 50 μg of antigen in Freund' complete adjuvant and were boosted at day 35 and day 70 with the same amount of antigen in Freund's incomplete adjuvant. Blood samples were taken at days 33, 68 and 103. Rabbit immunoglobulin was purified on Protein A Sepharose and buffer exchanged into PBS then concentrated to 20 mg/ml using Amicon Ultra centrifugal filters (Millipore).

### Parasite culture

*P. falciparum* asexual parasites were maintained in blood group O+ human erythrocytes at 4% hematocrit in RPMI-Hepes supplemented with 5% AlbumaxII (Gibco) and 5% heat denatured human serum.

### Immunoblotting

Proteins from synchronized early ring stage parasite culture supernatant were separated on 3–8% Tris-acetate SDS-polyacrylamide gels (Invitrogen) and transferred onto nitrocellulose (Invitrogen). Proteins were detected using rabbit serum primary antibody and horseradish peroxidase-conjugated goat anti-rabbit IgG secondary antibody (Dako). Blots were processed with an enhanced chemiluminescence system (ECL-Amersham).

### Assessment of antibody responses by cytometric bead array (CBA)

BD CBA Functional Beads (BD Bioscience, San Diego, CA, USA) of different non-overlapping fluorescence emission intensities were covalently coupled to *P. falciparum* recombinant proteins following the manufacturer's protocol. Briefly, 75 μl of selected micro-beads were sonicated for 1 min and then incubated with 1.9 µl of 1 M Dithiothreitol for 1h at 22°C with agitation. The beads were then washed 3 times and resuspended in 20 µl of Coupling Buffer (BD Bioscience). Prior to conjugation, 90 µg of *P. falciparum* proteins diluted at a final concentration of 1 mg/ml were activated by incubation with 2 µl of Sulfosuccinimidyl 4-N-maleimidomethyl cyclohexane 1-carboxylate (2 mg/ml) for 1 h. The protein mixture was then run through a buffer exchange spin column (Bio-Rad) pre-equilibrated with Coupling Buffer. The activated protein was added to the washed micro-beads and allowed to conjugate for 1 h at 22°C with agitation. Two µl of N-Ethylmaleimide (2 mg/ml) were added and the mixture was incubated for another 15 min. The beads were then washed, resuspended in 0.5 ml of Storage Buffer (BD Bioscience) and kept at 4°C, protected from the light until use.

For assessment of antibody responses, 1 µl of conjugated micro-beads was diluted in 50 µl of Washing Buffer (BD Bioscience) containing different dilutions of rabbit antibodies or non-immune rabbit sera. The antibodies (triplicates) were incubated for 30 min at 22°C, washed and then incubated with an anti-rabbit-IgG PE-conjugated antibody (BD, Bioscience). After washing, the samples were acquired using an LSR Fortessa analyzer (Becton Dickinson, New Jersey, USA). Analysis was performed using FlowJo software. The average mean fluorescent intensity (MFI) of non-immune serum samples +2SD was used as a cut-off value for calculation of end-point titres.

### Growth Inhibition Assay

A Growth Inhibition Assay (GIA) protocol was modified from a previously described method [38]. Briefly, 10 μl of trophozoite stage parasites were added to 35 μl of erythrocytes at 2% hematocrit in 96 well round bottom microtitre plates (Falcon) at either 0.5% parasitemia for one cycle of growth or 0.1% parasitemia for two cycles of growth then 5 μl of IgG or serum was added to each well. For two cycle assays, cultures were supplemented with 10 μl of media after 48 hours.

After incubation for either 48 hours (one cycle of growth) or 90 hours (two cycles of growth) each well was fixed at room temperature for 30 minutes with 50 μl of 0.25% glutaraldehyde (ProSciTech) diluted in PBS. Following centrifugation at 1200 rpm for 2 minutes, supernatants were discarded and trophozoite stage parasites were stained with 50 μl of 5X SYBR Green (Invitrogen) diluted in PBS. The parasitemia of each well was determined by counting 50,000 cells by flow cytometry using a Cell Lab Quanta SC – MPL Flow Cytometer (Beckman Coulter). Growth was expressed as a percentage of the parasitemia obtained using a pre-immune serum or IgG control. All samples were tested in triplicate.

GIA assays conducted at the MVI Reference Laboratory at NIH were performed as described previously [39]. This one-cycle assay was performed at a final IgG concentration of 0.016–2 mg/ml against the 3D7 *P. falciparum* clone.

### Estimation of IC_50_

The IC_50_ for each inhibitory antibody in growth inhibition assays was determined using Graphpad PRISM (Graphpad Software) following the recommended protocol for non-linear regression of a variable slope, four parameter, log(inhibitor) vs response curve.

### Inhibition of Invasion Assay

The inhibitory activity of antibodies against merozite invasion was directly determined using viable *P. falciparum* merozoites made from the D10-PfPHG line [40] as previously described [41]. Briefly, tightly synchronized late stage parasites (40–44 hours post invasion) were magnet purified away from uninfected RBCs and incubated with the protease inhibitor *trans*-Epoxysuccinyl-L-leucylamido (4-guanidino) butane (E64) for 6 hours. After centrifugation and resuspension in RPMI-HEPES the E64 treated schizonts were isolated by filtration through a 1.2 μm syringe filter (Acrodisc 32 mm, Pall). Purified merozoites (22.5 μl) were incubated with inhibitory antibodies (2.5 μl) for 10 minutes at 37°C. Uninfected RBCs (0.5% final) were then added and the culture agitated at 400 rpm for 10 minutes. Newly invaded ring stage parasitemia was assessed by flow cytometry (FACSCalibur) after 1 hour with the addition of 5 μg/ml ethidium bromide (no wash). Flow cytometry data was analyzed using Flow Jo (Tree Star Inc.) and ring stage parasite populations were counted using an Fl-1 high (GFP) and Fl-2 low (EtBr) gate.

### Immunofluorescence Microscopy

W2mef and W2mef175-Cterm truncation parasites were prepared as E-64 (trans-Epoxysuccinyl-L-leucylamido (4-guanidino) butane)-treated schizonts (ETSs) or invading merozoites [41]for immunofluorescence assay [42], and imaged using wide field deconvolution microscopy, as described previously [43]. Antibodies were diluted as follows: mouse anti-EBA175 RIII-V MAb 48/09-10F9-8-3 (1∶300), rabbit anti-EBA140 R1123 (1∶300), rabbit anti-AMA1 (1∶250) [17], rabbit anti-RON4 (1∶250) [44]. Goat anti-mouse and rabbit Alexa Fluor® 488 and 594 labeled secondary antibodies (Molecular Probes) were diluted 1∶500.

Zeiss Axiovision release 4.8 software (Zeiss) was used for fast-iterative deconvolution. General image processing was undertaken using ImageJ [45] and Adobe Photoshop CS5 software.

## Results

### Production and Immunization of Vaccine Candidate Antigens

**Figure 1:**
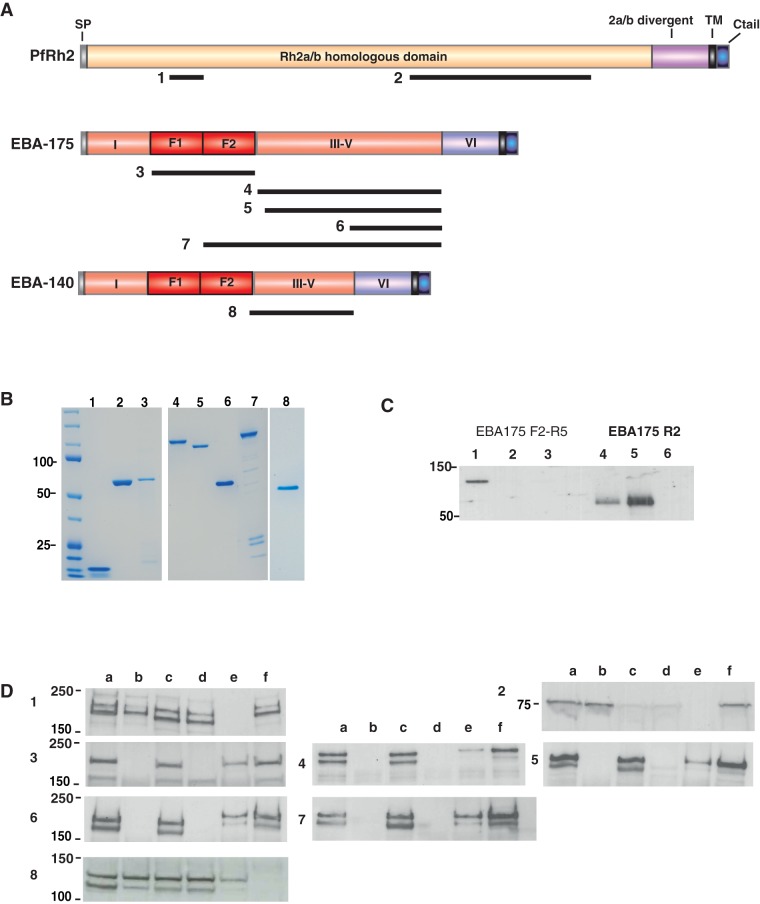
Antibodies raised in rabbits against recombinant *P. falciparum* invasion ligands specifically recognize native parasite antigens. **A**: Schematic of *P*. *falciparum* invasion ligands used to generate antigens for immunization. Black bars below the schematic structures indicate the regions of expressed recombinant proteins. Numbers indicate the lanes on the coomassie gel shown in fig. 1B. **B**: Antigenic fragments of invasion-related proteins were expressed and purified for rabbit immunization. Immunogens are shown here on SDS-PAGE. (1) Rh2 N-terminal fragment (2) Rh2 C-terminal fragment (3) EBA-175 Region 2 (4) EBA-175 Region 3-5 3D7 (5) EBA-175 RIII-V W2mef (6) EBA-175 RIV-V (7) EBA-175 F2-R5 (8) EBA-140 RIII-V. Proteins were stained with coomassie blue. **C**: Recombinant Region II of EBA-175 binds to erythrocytes in a sialic-dependent manner. Recombinant proteins EBA-175 F2-RV and RII were tested for binding to erythrocytes. Lanes 1 & 4 show protein pre-assay; 2 & 5, binding to untreated erythrocytes; Lanes 3 & 6, binding to neuraminidase-treated erythrocytes. **D**: Rabbit antibodies raised to recombinant antigens specifically recognize native parasite proteins. 1–8 correspond with antigens as described in Fig. 1A. Lanes as follows: 3D7 wild type (a), 3D7Δ175 KO (b), W2mef (c), W2mefΔ175 KO (d), FCR3 wild type (e), 3D7Δ140 KO (f).

A comparative study was undertaken to assess the relative potential of various malaria proteins, with known roles in erythrocyte invasion, for their ability to induce strain-independent inhibition of *P. falciparum* invasion. Eight antigens selected from 3 different *P. falciparum* proteins were expressed and purified for rabbit immunization studies (Fig. 1, Table S1). Two different regions of PfRh2 were expressed: a small N-terminal fragment recently identified as a minimal erythrocyte receptor-binding region and target of antibodies that inhibit merozoite invasion of erythrocytes [35] (Fig. 1B lane 1); and a larger fragment that in combination with EBA-175 antibodies enhanced growth inhibitory activity [13] (Fig 1B lane 2). Clinical grade EBA-175 Region II from the 3D7 strain (Fig. 1B lane 3) expressed in *Pichia pastoris* was kindly provided by Science Applications International Corporation (SAIC). Five other EBA-175 antigens were expressed in *E. coli*; and purified from the soluble fraction without refolding (Fig. 1B lanes 4–7). These represented both dimorphic alleles of RIII-V, RIV-V, and F2-RV from 3D7. EBA-140 RIII-V was also included in this antigen comparison (Fig. 1B lane 8).

Antigen structural integrity was confirmed by testing recombinant proteins in erythrocyte-binding assays where appropriate (Fig. 1C), incorporating neuraminidase treatment to remove sialic acid residues from surface receptors. EBA-175 RII bound erythrocytes in a sialic acid-dependent manner as expected. EBA-175 F2-RV (Fig. 1C) and both EBA-175 and EBA-140 RIII-V did not bind erythrocytes as expected (data not shown). To confirm recognition of native parasite proteins, sera were tested for reactivity against post-invasion culture supernatants or schizont lysates of various *P. falciparum* strains by western blot. All sera recognized native parasite proteins and were antigen specific (Fig. 1D). High titres of antibody were induced against all antigens, with endpoint titration of IgG ranging between 1/1,280,000 and 1/10,240,000 (Table 1).

**Table 1:** IC_50_ values for total IgG against different antigens in GIA.

Antigen	End-point Titre	End-point IgG Concentration ng/ml	IC50# 3D7 g/ml	IC50# W2mef g/ml	IC50# FCR3 g/ml	Average IC50 3 strains	
EBA-175 R3-5 (3D7)	1/1,280,000	16	9	30	<16	17	*Fig. 2A*
EBA-175 R3-5 (W2mef)	1/2,560,000	8	37	45	60	47	*Fig. 2C*
EBA-175 F2-R5 (3D7)	1/2,560,000	8	56	85	50	64	*Not shown*
EBA-175 R4-5 (3D7)	1/2,560,000	8	198	250	175	208	*Fig. 2H*
EBA-140 R3-5 (3D7)	1/5,120,000	4	200	1250	1250	900	*Fig. 4*
EBA-175 R2 (3D7)	1/1,280,000	16	NA	NA	NA	NA	
PfRh2-NTR rbc binding	1/10,240,000	2	NA	NA	NA	NA	
PfRh2-2A9 CTR	1/5,120,000	4	NA	NA	NA	NA	

# IC_50_ values were calculated by interpolation on the plot of total IgG[log_10_] versus % GIA with measured points connected by straight lines (as described in [56], calculations were performed on % GIA values in a 1-cycle assay, with IgG titrated from 2 mg/ml.

### EBA-175 Region III-V antibodies potently inhibit geographically and genotypically diverse parasite strains

**Figure 2:**
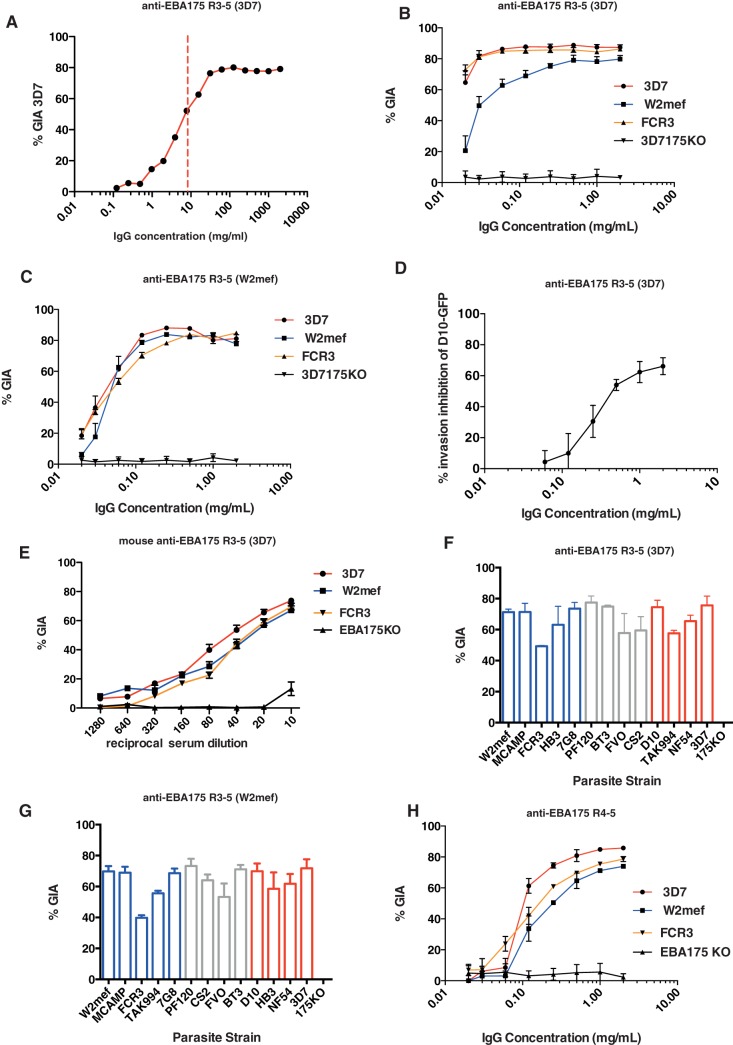
Epitopes of highly inhibitory, strain-transcending antibodies are located within the conserved region of EBA-175. (A) GIA against the homologous 3D7 parasite strain. Rabbits were immunized with 3D7 EBA-175 region III-V. IgG purified from rabbit serum was serially diluted from a starting concentration of 2 mg/ml. Data points indicate mean values of triplicate wells ± standard error of the mean (SEM) in a single assay relative to pre-immune IgG, calculated from a 1-cycle FACS-based assay. The dashed red line indicates the IC50 value of IgG (9 μg/ml). (B) Rabbit IgG against EBA-175 region III-V (3D7) – GIA against homologous and heterologous parasite strains. IgG from a single rabbit were tested against 3D7, W2mef, FCR3 and 3D7Δ175 KO parasite strains. Data points represent mean values of triplicates from 3 independent replicate experiments ± SEM in a 2-cycle assay. The antibody is completely specific to EBA-175 as there is no inhibition of the 175-KO parasites. (C) Rabbit IgG against EBA-175 region III-V (W2mef) – GIA against homologous and heterologous parasite strains. IgG from a single rabbit were tested against 3D7, W2mef, FCR3 and 3D7Δ175 KO parasite strains (2 cycle assay). (D) Anti- EBA-175 RIII-V directly inhibit invasion of purified, viable, D10-GFP merozoites. Results shown are mean + standard error of three independent experiments. (E) Mouse serum against EBA-175 region III-V (3D7) – GIA against homologous and heterologous parasite strains. Three individual mouse sera were pooled and tested against 3D7, W2mef, FCR3 and 3D7Δ175 KO parasite strains (1 cycle assay). (F) GIA patterns of Ig raised against both dimorphic alleles of EBA-175 against a panel of laboratory parasite strains are highly correlated. GIA was performed in a one cycle assay using anti-EBA175RIII-V (3D7) IgG at a concentration of 1 mg/ml. Blue histograms indicate parasite strains that invade predominantly via sialic-acid dependent pathway, red indicates sialic acid-independent pathway [63], grey bars where invasion preference is not known. Parasite strains belonging to the C-type group are shown in blue font whereas those belonging to the F-type are shown in red font. (G) GIA as for (F) but using anti-EBA175RIII-V (W2mef) IgG. (H) GIA using rabbit anti-EBA175 RIV-V (3D7) IgG (One-cycle assay).

A previous study indicated that rabbit immune serum against EBA-175 RIII-V (derived from the 3D7 strain) were around 20% inhibitory by growth inhibition assay (GIA) against the homologous parasite strain [13]. In that study, the antigen was produced in *E. coli* as an N-terminally tagged GST fusion protein. In order to comply with FDA regulations for human vaccine trials, we re-expressed this antigen as a fusion protein with a 6-HIS tag. Surprisingly, and in contrast with previous results using the same antigen as a GST-tagged fusion protein, high GIA activity of around 80% was observed against the 3D7 homologous parasite strain with IgG from rabbits immunized with the 6-His-tagged antigen. This remained potent at very low IgG concentrations with an IC50 for total IgG against RIII-V of 9μg/ml as calculated by interpolation on the x-axis of the point at which GIA  = 50% (Fig. 2A).

Because of the dimorphic nature of EBA-175 RIII-V, rabbit IgG was also generated against W2mef RIII-V representing C-type alleles of this protein (3D7 belonging to the F-type) to determine whether antigenic diversity between the two allelic types affected cross-strain protection. Surprisingly, both antibodies inhibited parasite growth of homologous and heterologous parasite strains by around 80% compared with control IgG, at antibody concentrations ranging from 0.5 to 2 mg/ml (Fig. 2B & C). The inhibitory capacity of antibodies raised against W2mef RIII-V appeared to titrate down to zero GIA activity at 16 μg/ml (Fig. 2C), whereas anti-3D7 RIII-V antibodies showed more than 60% inhibition against 3D7 and FCR3 parasites at a concentration of 16 μg/ml. W2mef parasites appeared to be less susceptible to growth inhibition compared to the other two strains, but nonetheless were still inhibited by 70% compared with control IgG at a concentration of 0.5 mg/ml (Fig. 2B). The inhibitory activity of the anti-EBA-175 RIII-V IgG was entirely specific to EBA-175, as no inhibition was observed against transgenic EBA-175 knock-out parasites. We tested the invasion inhibitory activity of antibodies raised against EBA-175 RIII-V (3D7) using purified *P. falciparum* merozoites and confirmed that these antibodies are potent inhibitors of merozoite invasion (Fig. 2D). To control for any specific inhibitory response restricted to rabbits, we also immunized mice with EBA-175 RIII-V. GIA confirmed that pooled mouse serum (not IgG purified) was also highly inhibitory to parasite growth in an antigen-specific and titratable manner against three parasite strains (Fig. 2E).

### Region IV-V of EBA-175 contains major epitopes for neutralizing antibodies

Having identified evidence for cross-strain immunity arising from RIII-V, we extended GIA analysis at a fixed concentration of 1 mg/ml against a panel of 13 independent *P. falciparum* parasite strains chosen on the basis of their global geographical origin and invasion pathway preference. A striking concordance was observed between the two antisera (IgG against 3D7 and W2mef EBA-175 RIII-V) in the pattern of GIA activity across these strains (Fig. 2 F&G), confirming that these antibodies neutralize parasite growth in a strain-independent manner. To confirm that sequence polymorphism did not affect GIA susceptibility, sequencing was performed across RIII-V for all strains tested. No polymorphisms were found within RIII sequences; 3D7, NF54, FCR3, BT3, TAK 994, FVO and CS2 were identical F-type alleles, and W2mef, MCamp, HB3, Pf120, 7G8 and D10 were identical C-type alleles. Within RIV-V, complete identity was found between W2mef and 3D7 and two identical amino acid changes found in all but one of the other strains (Table S2). Overall, the limited variation across strains in the target regions of the anti-EBA 175 RIII-V antibodies likely determines the ability of serum against this antigen to confer strain-independent growth inhibition. Furthermore, cross strain inhibitory profiles of antibodies raised against dimorphic forms of RIII-V indicated that the predominant growth inhibitory response was likely directed to the conserved RIV-V region. RIV-V was therefore expressed as a separate antigen and used to immunize rabbits to determine whether this shorter, conserved domain could induce cross-strain inhibitory antibodies. As predicted, IgG targeting the EBA-175 conserved RIV-V was highly inhibitory to parasite growth, with GIA ranging between 72–85% across 3D7, FCR3 and W2mef strains at 2 mg/ml (Figs. 2H). At lower antibody concentrations, anti-RIV-V IgG was less inhibitory than anti-RIII-V.

### Parasite Invasion pathway switching does not affect efficacy of growth inhibitory antibodies

**Figure 3:**
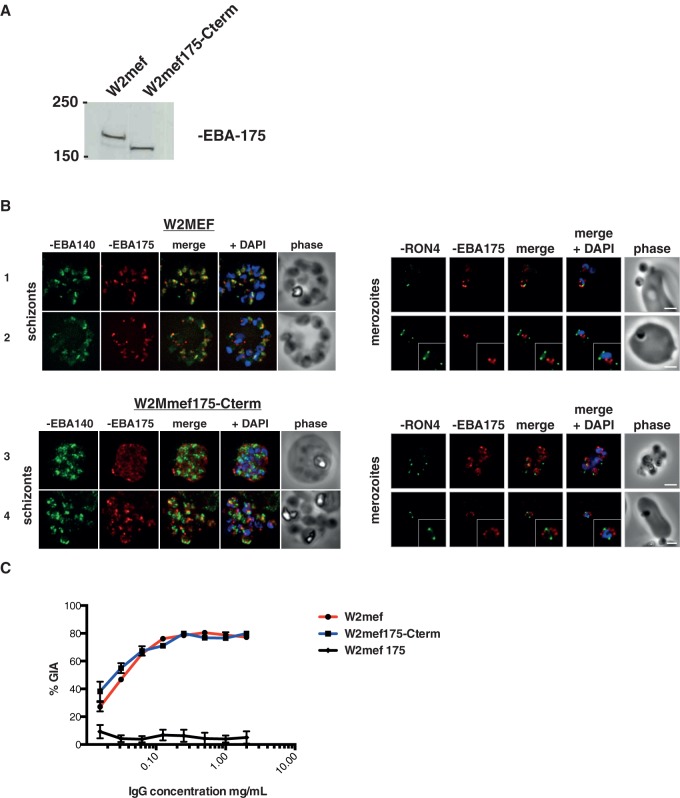
EBA-175 inhibitory antibodies block merozoite invasion through receptors other than Glycophorin A. (A) Western blot of post-invasion culture supernatants from W2mef and W2mef175-Cterm parasites probed with monoclonal antibody 9C4. This specifically recognizes EBA-175 region III-V and shows the truncated form of the protein that includes RIII-V was present in W2mef175-Cterm parasites. (B) EBA-175 requires motifs downstream of region 5 for correct trafficking within merozoites prior to invasion. Late segmented schizonts (ETS preparations) were co-stained with antiserum against EBA-140 to detect micronemes (green), along with inhibitory mAb targeting EBA-175 (red). Indirect immuofluorescence and phase contrast micrographs show correct co-localization within micronemes in wild type parasites (panels 1 &2) and mis-localization of truncated EBA-175 protein in transgenic parasites, often with aberrant, early secretion (panels 3&4). Free and invading merozoites, co-stained with the same W2mef-specific EBA-175 mAb (red) and RON4 to mark the tight junction (green), show that EBA-175 is present during merozoite invasion in both wild type and transgenic parasites. In all cases nuclei are stained with DAPI (blue) and scale bars show 2 μm. (C) W2mef and W2mef175-Cterm parasites were tested in GIA with IgG against EBA-175 RIII-V (3D7). Both parasite lines are inhibited equally efficiently by this IgG, whereas no inhibition is seen against EBA-175 KO parasites (One-cycle assay).

Whilst IgG raised to EBA-175 RIII-V was inhibitory to parasite strains that can use pathways other than EBA-175-Glycophorin A to invade, (e.g. 3D7 parasites preferentially invade erythrocytes using a sialic acid-independent pathway [46]), we were interested in testing whether a parasite that had switched to an alternative invasion pathway [47], [48] could influence the efficacy of a vaccine-induced immune response. We therefore tested a transgenic parasite W2mef175-Cterm, in which the *EBA-175* locus has been modified and expresses an EBA-175 protein truncated 35 amino acids upstream of the RV boundary. This has deleted the entire region RIV, the trans-membrane and cytoplasmic domains, but leaves region RIII-V of EBA-175 intact (Fig. 3A). Of note, these parasites are no longer capable of invading erythrocytes using EBA-175 and switch to a sialic acid independent pathway by transcriptional activation of PfRh4 [7], [47].

Immunoblot (Fig. 3A) and immunofluorescence microscopy (Fig. 3B) confirmed that this truncated EBA-175 protein was present in schizonts and merozoites, despite the loss of its C-terminal end that includes transmembrane anchor and cytoplasmic tail. In wild type W2mef parasites (expressing full-length EBA-175), immunofluorescence localization confirmed that EBA-175 co-localized with EBA-140 consistent with a micronemal location. In W2mef175-Cterm parasites, however, the truncated EBA-175 protein consistently failed to co-localize with EBA-140, confirming that RVI onwards encodes motifs that are essential for correct subcellular trafficking of EBA-175 [49]. The truncated protein frequently showed aberrant secretion during schizogony (Fig. 3B, panel 3). Nevertheless, imaging of merozoites, including those midway through invasion, showed that some EBA-175 was always present in both W2mef and W2mef175-Cterm parasites during invasion. Thus some truncated EBA-175 likely reaches the micronemes, potentially evident in areas of low-level signal coincidence between EBA-175 and EBA-140 in W2mef175-Cterm schizonts (Fig. 3 B panel 4).

By GIA, IgG against 3D7 EBA-175 RIII-V inhibited invasion of W2mef175-Cterm to the same level (80% inhibition) as W2mef wild type parasites (Fig. 3C). This indicates that antibody efficacy is unaffected by a switch in parasite invasion pathway usage or expression of a truncated version of EBA-175. W2mefΔ175 KO parasites, in which the entire *eba-175* gene is deleted [46] were not inhibited, further confirming antibody specificity for EBA-175.

### EBA-140 RIII-V induces growth inhibitory antibodies

**Figure 4:**
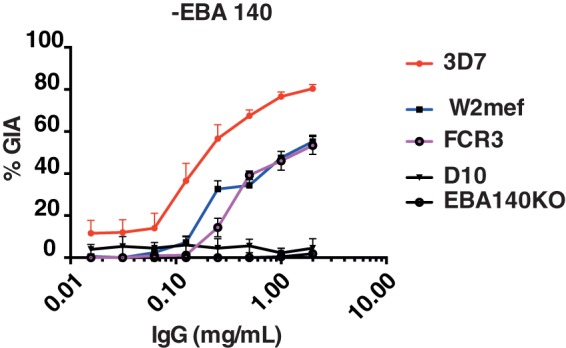
Antibodies targeting region 3–5 of EBA-140 also mediate cross-strain protection against *P. falciparum* IgG purified from immune rabbit serum was tested in GIA against 3D7, W2mef and FCR3 strains. No inhibition was observed against EBA-140 knock out parasites. Highest GIA levels were against the homologous strain at 2 mg/ml. Error bars represent SEM (One cycle assay).

To explore whether targeting RIII-V in other functional members of the PfEBA family of ligands had similar inhibitory effects on parasite growth, we expressed the same region from EBA-140, immunized rabbits and performed GIA against three *P. falciparum* strains, as described for EBA-175. Again, 3D7 was the strain most susceptible to IgG against EBA-140, with 80% inhibition, with W2mef and FCR3 less so. Both strains, however, still showed around 60% inhibition at 2 mg/ml IgG. Again, the inhibition observed was specific to EBA-140, since there was no effect against EBA-140 knockout parasites, or against D10, which has lost the *EBA-140* gene (Fig. 4). Thus RIII-V shows potent inhibitory potential against two and potentially all EBL proteins.

### High potency of IgG against conserved region of EBA antigens

**Figure 5:**
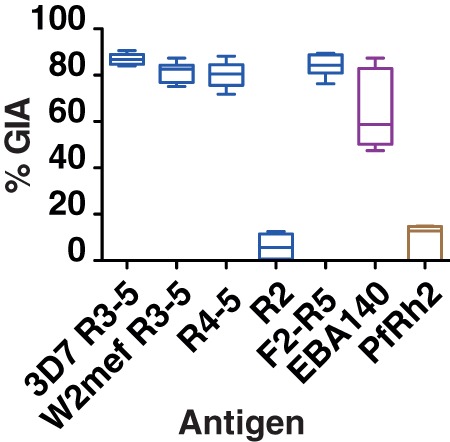
Summary of GIA data for antibodies tested against three strains of *P. falciparum*. Data is represented as box and whiskers plot; line represents the median inhibition of a particular antibody against the three strains 3D7, FCR3 and W2mef; box, interquartile range; and whiskers GIA minimum and maximum values. Data included in the analysis are derived from triplicate wells in 3 independent, 2-cycle assays with IgG at a concentration of 2 mg/ml. Anti-EBA-175 values in blue, anti-EBA-140 in purple and anti-Rh2 antibodies in brown. Error bars represent SEM.

Having established the potency of anti-RIII-V serum by GIA, we sought to select the best antigen partner for EBA-175 in an invasion-blocking combination vaccine, and test other potential EBA-175 candidate regions for suitability. Rabbit IgG from immune sera against a panel of other parasite invasion ligands (Fig. 1) were tested against 3D7, FCR3 and W2mef in GIA assays at a concentration of 2 mg/ml. Of the four non-EBA-175 antigens, only EBA-140 antibodies were over 50% inhibitory against all parasite strains (Fig. 5). The median % GIA across the three parasite strains was calculated for each antibody and this confirmed that antigens that included EBA-175 region IV-V induced the highest inhibition levels. Median inhibition for anti-EBA-140 RIII-V was lower than the EBA-175 antibodies, likely due to the reduced efficacy of EBA-140 antibodies against heterologous compared with homologous parasite strains, despite complete sequence conservation. GIA levels for IgG raised against the two PfRh2 antigens were similar to that found in previous studies with around 20% inhibition against 3D7 parasites (Fig. S1). The 3D7-EBA-175 knock-out line was the parasite strain most susceptible to inhibition by IgG targeting PfRh2, confirming previous findings that an additive or synergistic effect may be found between antibodies targeting EBA-175 and PfRh2.

**Figure 6:**
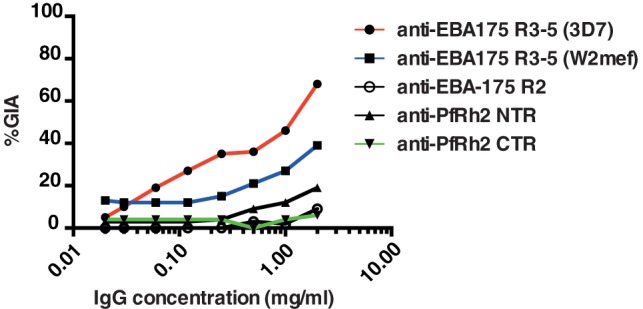
Data from independent testing of rabbit IgG for GIA activity. Data points indicate mean values of triplicate wells from a single-cycle pfLDH-based assay using 3D7 parasites. % GIA was calculated relative to pre-immune IgG.

These data clearly demonstrate that of all blood stage antigens tested in this study, EBA-175 region III-V performs best in a comparative GIA across different parasite genotypes. To confirm this finding, anti-EBA-175 RIII-V, anti-EBA175 RII and anti-Rh2 IgGs were tested independently in the GIA Reference Laboratory at NIH using 3D7 parasites. While there was a difference in absolute inhibition levels between the two sites, likely due to differences in study methodology, anti-EBA-175 RIII-V clearly out-performed anti-Region II antibodies, and anti-Rh2 N-terminal antibodies were significantly more inhibitory to parasites than anti-Rh2 C-terminal antibodies (Fig. 6).

An important consideration for vaccine efficacy against high numbers of transiently exposed blood stage parasites is that immunization should induce a potent antibody response. To examine the potency of inhibitory IgG against EBA-175 antigens, IC50 values were calculated. The numbers clearly show that not only are antibodies against EBA-175 conserved regions highly effective inhibitors of parasite growth at physiologically relevant IgG concentrations of 1–2 mg/ml, their activity remains high at low concentrations (Table 1). EBA-175 RIII-V (3D7) IgG was the most potent antibody tested with an average IC50 of 17 μg/ml across the 3 strains in our laboratory under our particular methodology.

## Discussion

Development of a blood stage vaccine against malaria has been hindered largely due to the high level of antigenic polymorphism in the few target antigens that have made it to the clinic, resulting in immune escape of parasites in both simian models [50] and clinical trials in human populations [51]. Results presented in this paper show that the conserved region of EBA-175 induces potent, parasite strain-independent, growth inhibitory antibodies that are active at low IgG concentrations. Further, we confirmed that antibodies raised against EBA-175 RIII-V inhibit merozoite invasion directly, rather than inhibiting intracellular parasite growth, in an inhibition of invasion assay using purified *P. falciparum* merozoites. We propose that EBA-175 RIII-V should be considered for further development as a component in a combination malaria vaccine.

In this head to head comparison of EBA-175 antigens, antibodies raised against RIII-V were more inhibitory to parasite growth than those against RII, and this was confirmed by independent testing at the GIA Reference Laboratory; although the overall potency of the antibody inhibition for the region III-V IgG was lower when tested there. One possible explanation for discrepancy in antibody potency between the two labs could be loss of antibody activity following freeze-thaw, since we have subsequently experienced the same phenomenon (Healer et al unpublished). This highlights the importance of standardized methodology for these types of studies in the future. EBA-175 RII induced weak growth-inhibitory antibodies against the homologous strain only, despite very good immunogenicity in rabbits. The reason RII did not induce highly inhibitory antibodies is unclear. One study that tested rabbit anti-RII antibodies in GIA reported high levels of invasion inhibition [52], but subsequent studies [32], [53], [54] report much lower levels of inhibition. This low level GIA may however be improved by synergy with antibodies targeting other merozoite antigens [55], [56]. A possible explanation for the differences in invasion inhibition of anti-RII IgG reported by different studies could be that the antigen preparations are expressed in different systems, and that protein folding, which is critical for epitope recognition is slightly different between different preparations. We confirmed that the RII antigen used in this study bound to erythrocytes in a sialic-acid dependent manner, which we assumed was indicative of correct structural conformation. Support for a role for RII in naturally acquired immunity to malaria is provided in a study of protective antibody responses in a cohort of semi-immune children in Papua New Guinea, which found a correlation between high levels of anti-EBA-175 RII IgG and RIII-V IgG and delayed time to clinical disease [12]. This however contrasted with previous studies in Western Kenya [10], [57] that found no association with antibody level against EBA-175 RII and clinical protection. More epidemiological studies, including a functional component, such as GIA, are needed across different geographical sites and with a range of antigens to further investigate such associations.

The testing of antibodies raised to two dimorphic alleles of EBA-175 RIII-V indicated that dimorphic region III contributes little to vaccine-induced immunity and conserved RIV-V contains the predominant epitopes of inhibitory antibodies. The central role of RIV-V in induction of functionally protective antibodies was confirmed by comparably high GIA with anti-RIV-V, RIII-V and F2-RV IgG. The F2-RV result also confirms that a conformationally intact F2 domain is not necessary for the induction of neutralizing antibodies targeting RIII-V. Why RIV-V antibodies were less inhibitory than anti-RIII-V at lower antibody concentrations is not understood. The end point IgG concentration measured against the immunizing antigens was in a similar range for both antibodies, however it was not possible to directly measure the specific antibody titre against native parasite protein, and it is a possibility that RIII when expressed in the context of RIII-V contributes to the conformation of neutralizing epitopes.

W2mef parasites were generally subject to lower levels of inhibition across the lower range of antibody concentrations even when the antibodies were generated with the W2mef RIII-V protein. This was somewhat unexpected, since W2mef is known to invade primarily via the EBA-175/Glycophorin A pathway, and so would be thought to be more susceptible to EBA-175 antibodies. This effect is not due to an invasion pathway switch or amino acid polymorphism. Possibly, EBA-175 protein concentration at the merozoite surface has some effect on susceptibility to inhibition at lower antibody concentrations. Nonetheless, the greater susceptibility of a supposedly sialic acid independent strain (3D7) at low antibody concentration indicates that invasion pathway preference does not determine susceptibility to antibody-mediated inhibition.

The high levels of polymorphism prevalent in RII, likely the result of strong immune-mediated diversifying selection on this erythrocyte binding region [29], may explain why antibodies against this region do not inhibit heterologous parasite strains, and this may contribute to the lack of association with protective immunity in some cohort studies, depending on which strain the antigen used in these studies originated from. In contrast, the high degree of sequence conservation in RIV-V suggests that it may be functionally constrained and therefore important for EBA-175 function or hidden from immune pressure to diversify. It is possible that the dimorphic region III of EBA-175 may be immunologically dominant, deflecting immune responses away from the functionally important RIV-V. Haplotype analysis of regions II, III and IV-V in clinical isolates of *P. falciparum* will be important towards determining whether other polymorphisms are present in field isolates, and sero-epidemiological analyses to elucidate the relative immunogenicity of these regions in malaria-exposed individuals will be necessary to address these important questions.

Further evidence that RIV-V is a key domain in the function of EBA proteins is clear from antibodies raised to the same region of EBA-140, which are also inhibitory to parasite invasion. EBA-140 is not a functional invasion ligand in W2mef and FCR3 parasites [58], which may explain the reduced susceptibility to anti-EBA140 antibodies by these parasite strains compared to 3D7. All three strains are genetically identical in region III-V of EBA-140, ruling out the possibility that strain-specificity is the reason for the reduced susceptibility to antibody-mediated inhibition seen in W2mef and FCR3. Naturally acquired antibodies to EBA-140 RII and RIII-V were also associated with protective immunity in children [12], strengthening the case for further vaccine-related research on this antigen. These findings warrant more research into the applicability of EBA-140 as a malaria vaccine candidate.

A recent paradigm for selection of vaccine candidates is that target antigens should be essential to parasite development, and so the ability to create *in vitro* genetic deletions of EBA-175 in two laboratory isolates would appear to rule out this ligand as a good vaccine candidate. In defense of EBA-175 however, there appears to be strong selection maintaining the presence of this gene in wild parasite populations, as it is the primary invasion ligand in many endemic parasite isolates [59], [60]. Expression levels of EBA-175 vary in lab and field isolates, but no parasite has been found in the field that does not express EBA-175 and no correlation has been found between varying expression level and invasion pathway usage [61].

The diversity of ligand-receptor interactions employed by *P. falciparum* provides a potential mechanism by which parasites evade antigen-specific inhibitory immune responses, and this has been demonstrated for antibodies targeting EBA-175 to some extent by comparing wild type and EBA-175-null parasites to demonstrate differences in susceptibility to naturally acquired growth-inhibitory antibodies in malaria exposed children [11]. We ruled out the possibility that vaccine induced antibodies targeting EBA-175 RIII-V might be restricted to parasite strains using an EBA-175 dependent, Glycophorin A invasion pathway, by using a transgenic line which expresses an inactive, truncated EBA-175 protein to demonstrate that vaccine-induced IgG inhibit invasion of this parasite strain as well as the wild type parasite. How this truncated protein retains its presence at the merozoite surface during invasion, despite some mis-localization compared to wild-type protein during schizogony, is not yet apparent though it is suggestive that EBA-175 may be part of a larger protein complex that traffics it along with other adhesins to the point of merozoite attachment with or without the cysteine-rich RVI domain. Irrespective of this, it is clear that antibodies targeting EBA-175 prevent merozoite invasion of erythrocytes independently of EBA-175/Gycophorin A pathway usage and despite the presence of other ligands that are expressed and functional in these parasites. Thus ligation of EBA-175 with an inhibitory antibody appears to prevent invasion through other alternative adhesin-dependent pathways. This finding was interesting in the light of data from an elegant study that examined the dynamics of release of different invasion ligands from apical organelles during merozoite invasion. It revealed that the EBA proteins (175 and 140) were translocated to the merozoite surface concurrently, and prior to exocytosis of PfRh2b and another rhoptry protein CLAG 3.1. Furthermore, it was found that receptor engagement by EBA-175 was required for release of rhoptry proteins [62]. We speculate that antibodies against EBA-175 RIII-V prevent release of rhoptry proteins indirectly, perhaps through prevention of RII engagement with glycophorin A.

The demonstration that growth inhibition by anti-EBA 175 antibodies is independent of invasion pathway is not novel, as studies with antibodies targeting RII have shown similar effects against strains using sialic-acid independent invasion [53], [54]. The levels of growth inhibition in those studies were, however, much lower than found here. Crucially though, the potency of RIII-V indicates that following vaccination with this antigen, the highly inhibitory induced immune response would not be circumvented by parasites switching invasion pathway. This indicates that vaccination with this antigen is unlikely to drive immune escape by mutation selection or switching invasion pathway since these mechanisms would be unlikely to provide an adaptive advantage to these parasites.

The finding that reconfiguring antigens by replacement of GST with histidine tags for expression and purification appeared to have a major effect on the production of inhibitory antibodies in rabbits was surprising, and true in the case of both EBA-175 and EBA-140 RIII-V, which had previously generated only mildly inhibitory antibodies after multiple immunizations in rabbits. It is possible that the larger GST tag may sterically mask neutralizing epitopes in these particular antigens, or perhaps more likely, the GST-tagged proteins used in earlier studies were not as conformationally representative of the native antigen.

Although we observed up to 90% inhibition of parasite growth, there was nonetheless variability between parasite strains in their susceptibility to antibody-mediated inhibition. This emphasizes the need to test many isolates in vaccine-related studies to gauge the potential global efficacy of candidate antigens. Our present findings indicate that EBA-175 RIII-V could be a key component in a future malaria vaccine, but would not successfully prevent all growth of all parasite strains. It has been suggested that an effective blood stage vaccine should include more than one antigenic target to cover potentially confounding effects of parasite phenotypic diversity. Previously, we saw elevated GIA levels, indicative of a synergistic effect on parasite growth when EBA-175 RIII-V was co-immunized with PfRh2 [13]. It now becomes an imperative to investigate the potentially synergistic interactions between antibodies targeting EBA and PfRh proteins in co-immunization studies towards full development of a strain-independent malaria vaccine.

## Supporting Information

Figure S1 **Antibodies against PfRh2 N-terminal fragment are superior to anti- C-terminal antibodies in GIA.** Rabbit IgG against PfRh2 antigens - GIA against homologous and heterologous parasite strains. IgG raised against Rh2 N-terminal fragment (A) and C-terminal fragment (B) were tested against 3D7, W2mef, FCR3 and 3D7Δ175 KO parasite strains. Data points represent mean values of triplicates from a 2-cycle assay.
(DOC)

Table S1 ***P. falciparum*** **blood-stage antigens tested in this study.**
(DOCX)

Table S2 **High Conservation across Sequences of EBA-175 RIII-V among diverse parasite isolates.**
(DOCX)
