# Supplementary material for: Vaccination with Conserved Regions of Erythrocyte-Binding Antigens Induces Neutralizing Antibodies against Multiple Strains of Plasmodium falciparum
Source: PLoS One. 2013 Sep 10;8(9):e72504. doi: 10.1371/journal.pone.0072504 (PMC3769340; doi:10.1371/journal.pone.0072504)
Supplement: Figure S1 — Antibodies against PfRh2 N-terminal fragment are superior to anti- C-terminal antibodies in GIA. Rabbit IgG against PfRh2 antigens - GIA against homologous and heterologous parasite strains. IgG raised against Rh2 N-terminal fragment (A) and C-terminal fragment (B) were tested against 3D7, W2mef, FCR3 and 3D7Δ175 KO parasite strains. Data points represent mean values of triplicates from a 2-cycle assay. (DOC) [file pone.0072504.s001.doc]

**Figure S1. Antibodies against PfRh2 N-terminal fragment are superior to anti- C-terminal antibodies in GIA.**


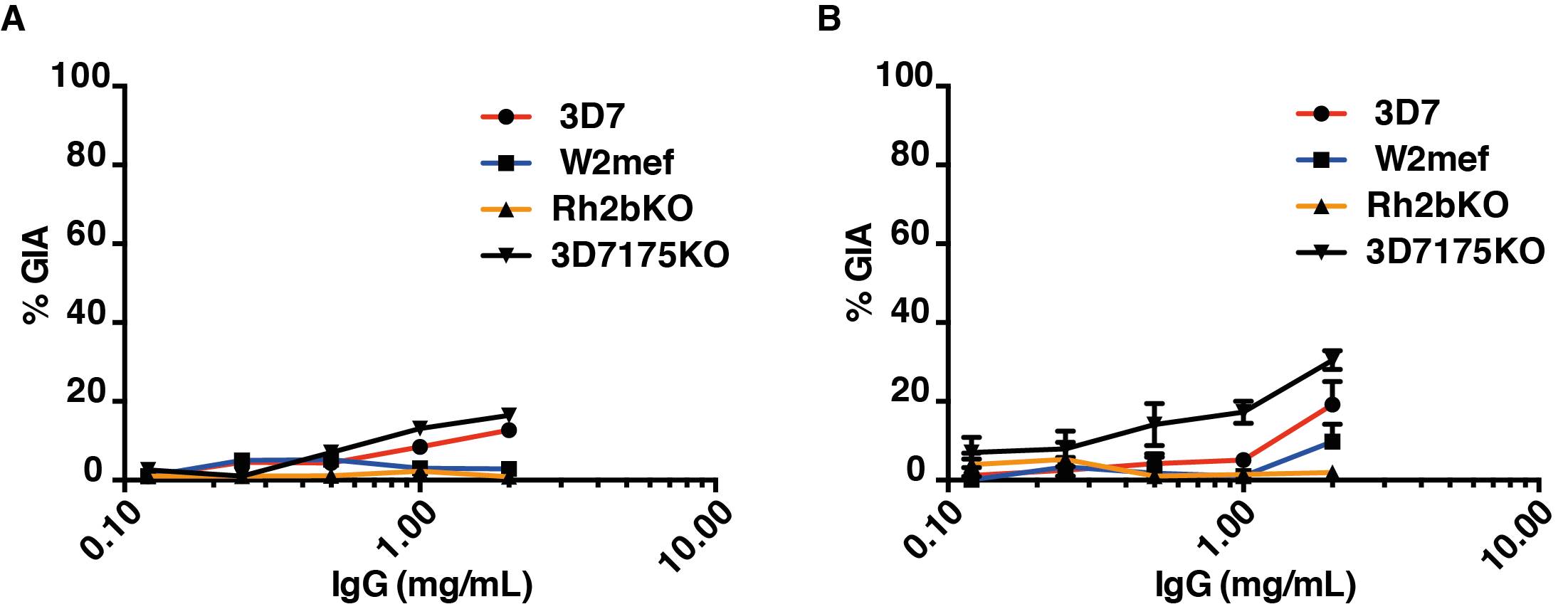


Rabbit IgG against PfRh2 antigens - GIA against homologous and heterologous parasite strains**.** IgG raised against Rh2 N-terminal fragment (A) and C-terminal fragment (B) were tested against 3D7, W2mef, FCR3 and 3D7 EBA-175 KO parasite strains. Data points represent mean values of triplicates from a 2-cycle assay.
