# Supplementary material for: Vaccination with Conserved Regions of Erythrocyte-Binding Antigens Induces Neutralizing Antibodies against Multiple Strains of Plasmodium falciparum
Source: PLoS One. 2013 Sep 10;8(9):e72504. doi: 10.1371/journal.pone.0072504 (PMC3769340; doi:10.1371/journal.pone.0072504)
Supplement: Table S1 — P. falciparum blood-stage antigens tested in this study. (DOCX) [file pone.0072504.s002.docx]

**Table S1.** *P. falciparum* Blood-Stage Antigens Tested in this Study.

| Ligand | Domain | Antigenic region | Parasite Strain | Expression System | Expression Tag | Lane  Fig. 1B |
| --- | --- | --- | --- | --- | --- | --- |
| PfRh2 | RBC-binding | 445-557 | 3D7 | *E. coli* | N-term 6HIS | 1 |
|  | 2A9 (Duraisingh et al) | 2033-2528 | 3D7 | *E. coli* | N-term 6HIS | 2 |
| EBA-175 | Region II | 145-760 | 3D7 | *Pichia* | none | 3 |
|  | Region III-V | 761-1298 | 3D7 | *E. coli* | N-term 6HIS | 4 |
|  | Region III-V | 761-1271 | W2mef | *E. coli* | N-term 6HIS | 5 |
|  | Region IV-V | 1010-1298 | 3D7 | *E. coli* | N-term 6HIS | 6 |
|  | F2-RV | 460-1298 | 3D7 | *E. coli* | C-term 6HIS | 7 |
| EBA-140 | Region III-V | 746-1043 | 3D7 | *E. coli* | C-term 6HIS | 8 |
