# Supplementary material for: Vaccination with Conserved Regions of Erythrocyte-Binding Antigens Induces Neutralizing Antibodies against Multiple Strains of Plasmodium falciparum
Source: PLoS One. 2013 Sep 10;8(9):e72504. doi: 10.1371/journal.pone.0072504 (PMC3769340; doi:10.1371/journal.pone.0072504)
Supplement: Table S2 — High Conservation across Sequences of EBA-175 RIII-V among diverse parasite isolates. (DOCX) [file pone.0072504.s003.docx]

**Table S2.** High Conservation across Sequences of EBA-175 RIII-V among diverse parasite isolates

| **Parasite line** | **origin** | **R3 dimorphic type** | **Start RIV 1010** | **1058** | **1100** | **End RV**  **1298** |
| --- | --- | --- | --- | --- | --- | --- |
| 3D7 | clone of NF54 | F | N | E | G | D |
| NF54 | ? Netherlands | F |  |  |  |  |
| FCR3 | Africa | F |  | V | D |  |
| TAK994 | SE Asia | F |  | V | D |  |
| CS2 | S. America - der. ItG2 | F |  | V | D |  |
| FVO | Vietnam | F |  | V | D |  |
| BT3 | PNG | F |  |  | D |  |
| D10 | PNG - clone of FC27 | C |  | V | D |  |
| 7G8 | Brazil | C |  | V | D |  |
| PF120 | PNG | C |  | V | D |  |
| HB3 | Honduras | C |  | V | D |  |
| MCAMP | SE Asia | C |  | V | D |  |
| W2mef | Indochina | C | N | E | G | D |
